# Supplementary material for: A high-quality reference genome for the common creek chub, Semotilus atromaculatus
Source: G3 (Bethesda). 2023 Dec 21;14(2):jkad283. doi: 10.1093/g3journal/jkad283 (PMC10849318; doi:10.1093/g3journal/jkad283)
Supplement: jkad283_Supplementary_Data [file jkad283_supplementary_data.zip › Supplemental_Figures_G3-2023-404422.pdf]

Supplemental Figures

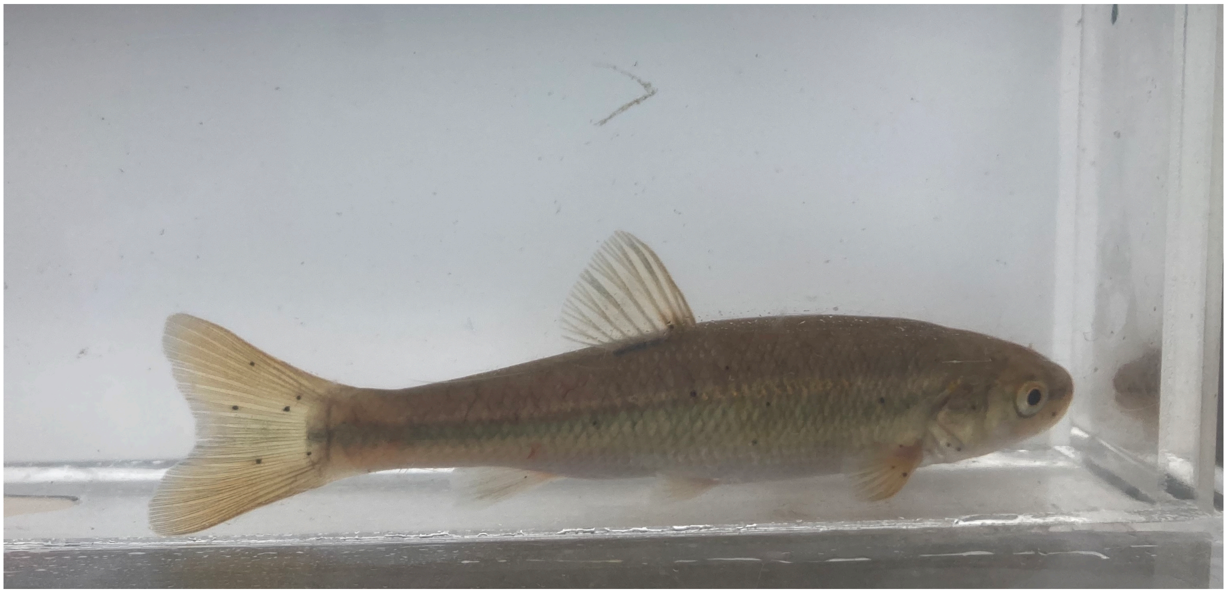

Figure S1: The creek chub individual used to create the reference genome. This fish was sampled from Swan Creek, Ontario, Canada. Note the dot present at the base of the dorsal fin and intermediate scale size compared to similar species. Not visible in the photo are the small barbels in the groove of each side of the mouth and minimally visible is the large mouth and black “moustache”.

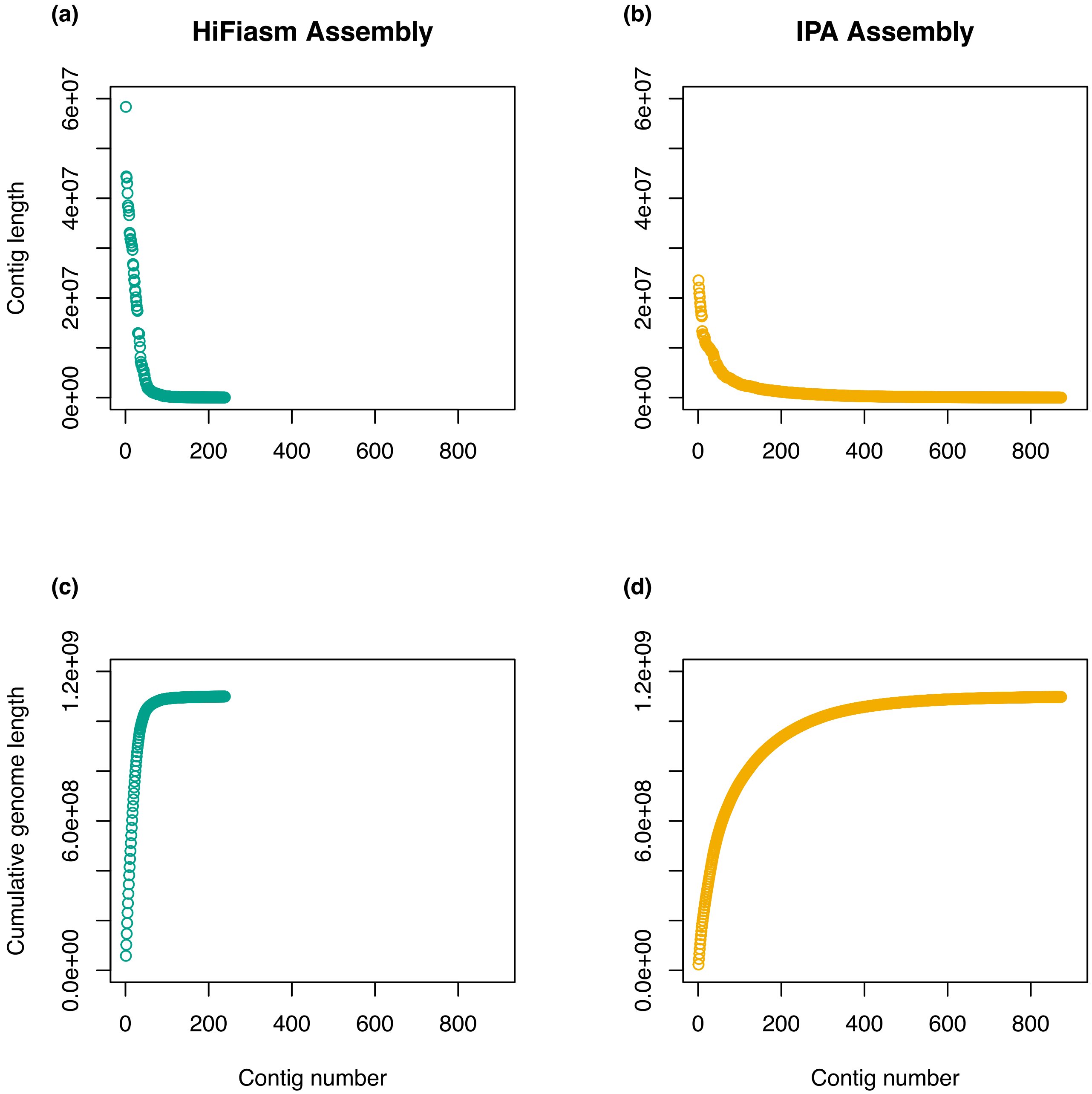

Figure S2: Visual comparison of the number of contigs, contig lengths, and cumulative genome lengths for both the HiFiasm and IPA genome assemblies. (a) Length of each contig in the HiFiasm assembly. (b) Length of each contig in the IPA assembly. (c) Cumulative genome length of HiFiasm assembly. (d) Cumulative genome length of IPA assembly.

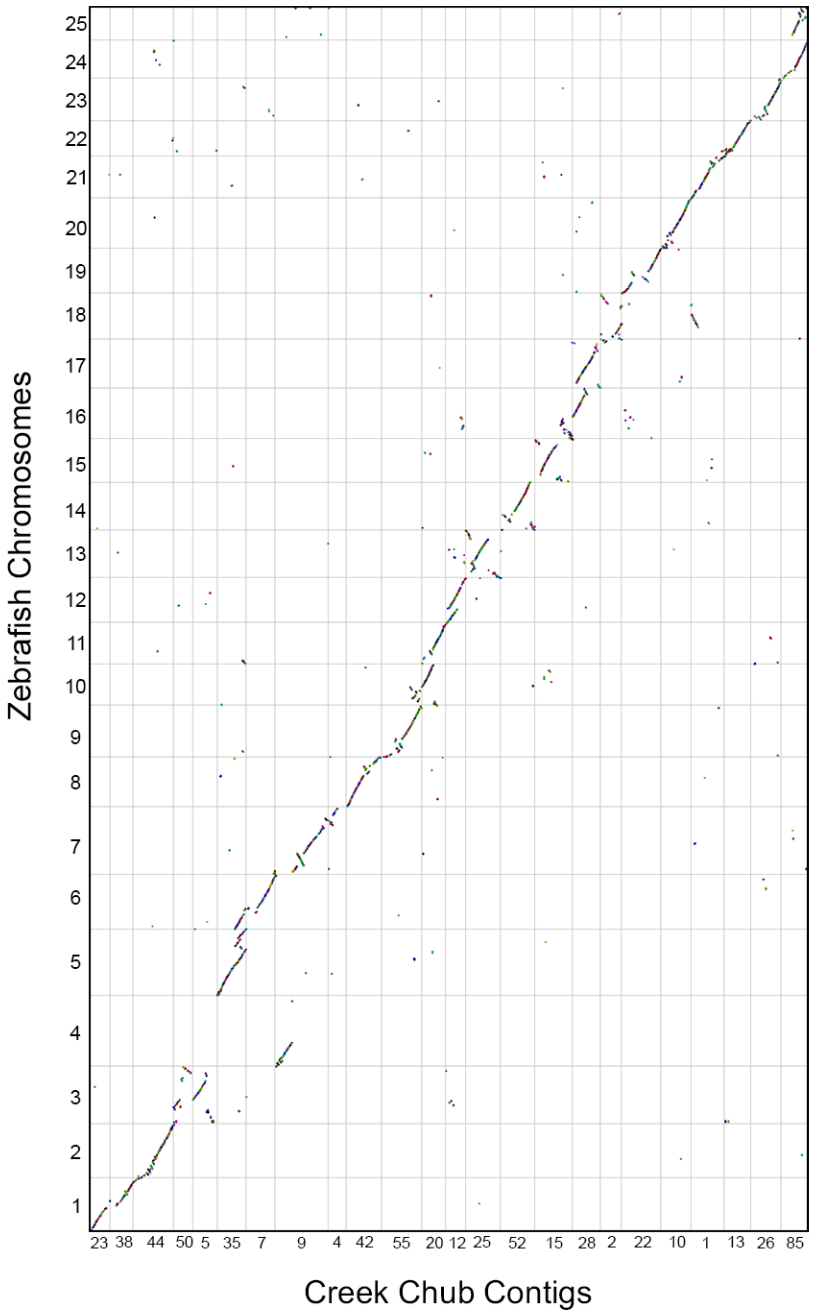

Figure S3: Dot plot made using CoGe's SynMap (Lyons & Freeling 2008) showing synteny between creek chub (x-axis) and zebrafish (y-axis). All 25 zebrafish chromosomes are present, while only the 25 largest contigs from the creek chub have been displayed, by setting the minimum chromosome length to 20,130,130 base pairs. Each colour represents a different syntenic block. The figure can be regenerated at any time by following this link: `genomevolution.org/r/1oxpw`

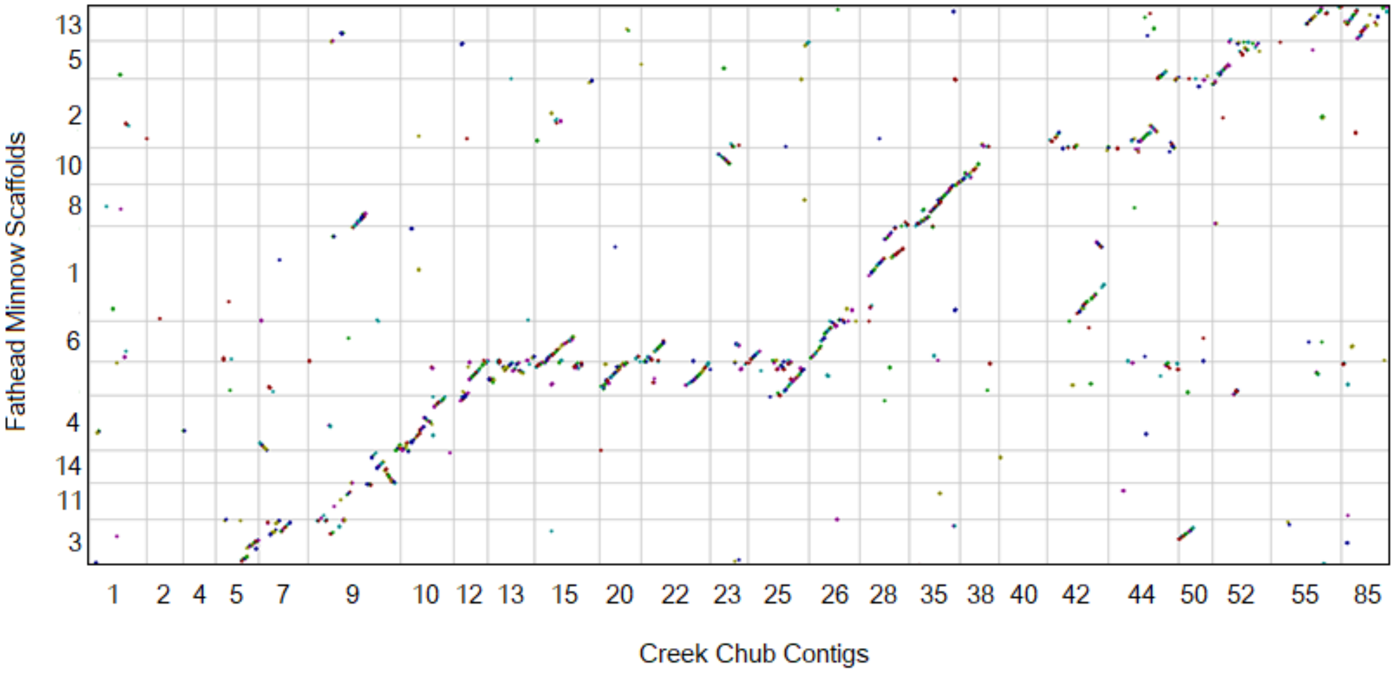

Figure S4: Dot plot made using CoGe's SynMap (Lyons & Freeling 2008) showing synteny between creek chub (x-axis) and fathead minnow (y-axis). Only the 25 largest contigs from the creek chub genome have been displayed, by setting the minimum contig length to 20,130,130 base pairs. Each colour represents a different syntenic block. The figure can be regenerated at any time by following this link: `genomevolution.org/r/1oxq3`
